# Synthesis of Magneto-Controllable Polymer Nanocarrier Based on Poly(N-isopropylacrylamide-co-acrylic Acid) for Doxorubicin Immobilization

**DOI:** 10.3390/polym14245440

**Published:** 2022-12-12

**Authors:** Viktoria S. Kusaia, Elena Yu. Kozhunova, Darya A. Stepanova, Vladislava A. Pigareva, Andrey V. Sybachin, Sergey B. Zezin, Anastasiya V. Bolshakova, Nikita M. Shchelkunov, Evgeny S. Vavaev, Evgeny V. Lyubin, Andrey A. Fedyanin, Vasiliy V. Spiridonov

**Affiliations:** 1 Department of Chemistry, Lomonosov Moscow State University, Leninskie Gory 1-2, 119234 Moscow, Russia; 2 Department of Physics, Lomonosov Moscow State University, Leninskie Gory 1-3, 119991 Moscow, Russia

**Keywords:** microgel, magnetosensitivity, controlled delivery, optical tweezers, static light scattering, model cell membranes, doxorubicin

## Abstract

In this work, the preparation procedure and properties of anionic magnetic microgels loaded with antitumor drug doxorubicin are described. The functional microgels were produced via the in situ formation of iron nanoparticles in an aqueous dispersion of polymer microgels based on poly(N-isopropylacrylamide-co-acrylic acid) (PNIPAM-PAA). The composition and morphology of the resulting composite microgels were studied by means of X-ray diffraction, Mössbauer spectroscopy, IR spectroscopy, scanning electron microscopy, atomic-force microscopy, laser microelectrophoresis, and static and dynamic light scattering. The forming nanoparticles were found to be β-FeO(OH). In physiological pH and ionic strength, the obtained composite microgels were shown to possess high colloid stability. The average size of the composites was 200 nm, while the zeta-potential was −27.5 mV. An optical tweezers study has demonstrated the possibility of manipulation with microgel using external magnetic fields. Loading of the composite microgel with doxorubicin did not lead to any change in particle size and colloidal stability. Magnetic-driven interaction of the drug-loaded microgel with model cell membranes was demonstrated by fluorescence microscopy. The described magnetic microgels demonstrate the potential for the controlled delivery of biologically active substances.

## 1. Introduction

Nowadays, magnetic nanoparticles have opened up a wide spectrum of different applications in bio- and nanomedicine. So far, magnetic nanoparticles have been used for magnetic separation (for the nucleic acid and cell separation) techniques [1,2], as contrast agents in magnetic resonance imaging [3], for local hyperthermia [4], for cell mechanics [5], for tumor progression [6], and for the in vivo tracking of stem cells [7]. As for biomedical applications, magnetic nanoparticles provide the perfect model for a high level of accumulation in the target tissues or organs due to their host cell tropism. However, it is crucial to choose suitable magnetic nanoparticles for the construction of nanodevices with adjustable chemical properties, i.e., the magnetic nanoparticles should be nontoxic. To this end, magnetic iron-oxide nanoparticles have become promising candidates, and they have been used for in vitro diagnostics. Among various types of iron-oxide magnetic materials, the nanoparticles based on the completely oxidized form of iron, maghemite (γ-Fe_2_O_3_), are by far the most commonly employed nanomaterial for biomedical applications. Another type of completely oxidized form of iron with magnetic properties is β-FeOOH. Due to their adjustable size, biocompatibility, and superparamagnetic behavior, one of the most important applications of magnetic nanoparticles is the creation of magnetically controlled carriers for drug delivery.

Being loaded by magnetic vehicles, the pharmaceutical drugs can be released at the target site with an external localized magnetic field gradient, thereby significantly reducing nonspecific toxicity by administering lower but more accurately targeted doses [8]. Due to these reasons, drug-loaded nanocarrier-based cancer therapy possesses the potential to overcome the toxicity of the drug and poor control over dosing when custom combination therapies are employed. The chemotherapeutic agents selectively targeting tumor cells can provide more effective cancer therapy.

Water-soluble in the protonated form, doxorubicin (DOX) is one of the most commonly used anticancer drugs for the treatment of many cancer types [9]. A significant disadvantage of doxorubicin is its strong cytotoxicity to normal tissues, including cardiac toxicity [10]. To reduce the cardiac toxicity of the free drug and alleviate the multidrug resistance effect, micelles and liposomes have been designed as delivery vehicles to encapsulate DOX noncovalently. Doxorubicin can also be covalently immobilized, e.g., for this purpose, gold nanoparticles were used [11]. To decrease the toxicity of DOX, the targeted delivery of the drug through macromolecules and polymer microgels is an alternative approach to cancer therapy [12,13].

Microgels, solvent-swollen networks present as discrete particles ranging from 20 nm to 50 µm in diameter, have been widely used as a promising material for drug delivery [14]. Microgels have the benefits of easy synthesis and control over important characteristics such as size, long-term stability, biocompatibility, biodistribution, bioaccumulation, and degradation. Furthermore, smart microgels based on poly(oligo(ethylene glycol) methacrylate) [15], poly(N-vinylcaprolactam) [16], or poly(N-isopropylacrylamide) [17] possess a lower critical solution temperature [18].

Microgels have many advantages as frameworks as they can be constructed in a broad range of sizes (from nanometers to micrometers), morphologies, and responsiveness to various stimuli [19]. A significant feature of polymer microgels is functionality. This term can be considered both in the context of drug immobilization and the stabilization of magnetic nanoparticles for the aim of constructing smart drug-loaded magneto-sensitive devices. Preparation of the composite microgels with incorporated magnetic nanoparticles could allow one to construct the nanocarrier with controllable transport and release properties under the impact of an external magnetic field [20].

In this work, we propose a simple approach for the preparation of a magnetic microgel based on poly(N-isopropylacrylamide–co-acrylic acid) (PNIPAM-PAA) filled with antitumor component DOX. The one-step in situ synthesis of β-FeOOH magnetic nanoparticles stabilized by PNIPAM-PAA microgel was used. The chemical structure of the inorganic nanoparticles was determined using Mössbauer spectroscopy. By means of atomic-force microscopy and Cryo-TEM, the morphology of magneto-modified microgel particles was established. Magnetic measurements with optical tweezers gave the cumulative magnetic moment of the individual NIPAM-PAA-β-FeOOH microgels. The filling of magnetic microgels with DOX was carried out and confirmed by means of IR and UV spectroscopy. The hydrodynamic and electrokinetic characteristics of ternary systems PNIPAM-PAA-β-FeO(OH)-DOX were analyzed. Demonstration of the magneto-controlled delivery of microgels into the biological membranes was made.

## 2. Materials and Methods

N-isporopylacrylamide (NIPAM—monomer, Sigma-Aldrich, St. Louis, MO, USA), N,N’-methylenebisacrylamide (BIS—crosslinking agent, Sigma-Aldrich), ammonium persulfate (AP—initiator, Sigma-Aldrich, St. Louis, MO, USA), and sodium dodecylbenzenesulfonate (SDBS—surfactant, Sigma-Aldrich, St. Louis, MO, USA) were used as received. Acrylic acid (AA—monomer, Sigma-Aldrich) was purified by distillation. Mohr salt (NH_4_)_2_Fe(SO_4_)_2_ × 5H_2_O (Merck, St. Louis, MO, USA), NaH_2_PO_2_ (Reachem, Moscow, Russia), NaOH (Reachem, Moscow, Russia), 2-hydroxy-5-sulphobenzoic acid (Sigma-Aldrich, St. Louis, MO, USA), and doxorubicin hydrochloride(Teva Pharmaceutical Industries, Tel Aviv, Israel) were used as received. Water was purified using a Millipore Milli-Q system.

PNIPAM-PAA microgels were synthesized by surfactant-assisted precipitation polymerization in water [21]. Polymerization was carried out under a nitrogen atmosphere in a glass reactor equipped with a thermostatically controlled chamber and a magnetic stirrer. In a typical procedure, 3.17 g of NIPAM, 0.87 g of AA, 0.06 g of BIS, and 0.05 g of SDBS were dissolved in 100 mL of water and placed into the reaction vessel. The solution was stirred (700 rpm) at 60 °C for 60 min under the nitrogen flow to allow complete dilution and oxygen removal. Then, ammonium persulfate was added under continuous stirring to start the reaction. The polymerization was carried out for 6 h, and then, the solution was cooled down slowly and purified by dialysis against deionized water (14 kDa MWCO) for 7 days. According to element analysis, the rate of C:N:H was 57.85:9.20:8.92.

The nanocomposites were prepared using the approach developed earlier for the synthesis of magnetic materials composed of magneto-ordered Fe(III)-oxide nanoparticles with oligo- and polysaccharides [22]. Microgel powder (10 mg) was dissolved in 10 mL of bidistilled water. Then, aqueous solutions (1 mL each) containing 7.5 mg of (NH_4_)_2_Fe(SO_4_)_2_ × 5H_2_O, 18 mg of NaOH, and 14 mg of sodium hypophosphite, respectively, were added dropwise to the resulting liquid. The resulting mixture was vigorously stirred. The end of the reaction was determined by the change from the green color of the mixture to dark red. The obtained solution was dialyzed against bidistilled water (“Sigma-Aldrich” dialysis sacks with MWCO ~ 14,000 kDa were used) to purify the polymer composite from dissolved low molecular weight compounds. Lyophilic drying was used to isolate the product.

Iron content was determined as described, for example, in our previous work [22], namely, spectrophotometrically with Ultrospec 4050 (LKB, Sweden). The principle of determination is based on the fact that Fe^3+^ forms a red–violet complex with sulfosalicylic acid at pH 1.8–2.5, characterized by maximum absorption at the wavelength of 510 nm. Firstly, the iron-containing particles were transferred to an ionized form by treating them with concentrated sulfuric acid; 5 mg of the lyophilized nanocomposite was dissolved in 0.1 mL of 10 mM H_2_SO_4_ aqueous solution and mixed with 5 mL of 10% 2-hydroxy-5-sulphobenzoic acid aqueous solution. Absorbance at 510 nm was registered. Using Formula (1), η was calculated.

η = (C × n × V × M)/(1000 × m) × 100%(1)

C is the concentration of Fe^3+^ ions (mol L^−1^), n is the degree of dilution of solutions, M is the molar mass of iron (56 g mol^−1^), and m is the mass of the composite (g).

The synthesis of DOX-loaded microgel was performed as follows. Iron-containing microgel powder (5 mg) was dissolved in 5 mL of bidistilled water. Then, 1 mL of an aqueous solution containing 0.25 mg of doxorubicin was added dropwise. The resulting mixture was vigorously stirred. The obtained solution was dialyzed against bidistilled water (“Sigma-Aldrich” dialysis sacks with MWCO ~ 14,000 kDa were used) to purify the polymer composite from dissolved low molecular weight compounds. Lyophilic drying was used to isolate the product.

The model lipid membrane was made by the formation of a lipid bilayer on the surface of 4.9 ± 0.5 μm borosilicate microspheres (BSMs, Duke Scientific, Paolo Alto, CA, USA). The procedure was carried out in two stages. In the first stage, the surface of the BSMs was cleaned by suspending the microspheres in methanol, with further separation by centrifugation, followed by resuspension in a 1 M KOH solution. Precipitated BSMs were rinsed five times with bidistilled water. At the second stage, BSMs (2 mg) were mixed with a 0.2 mg/mL suspension of anionic liposomes from dioleoylphosphatidylglycerol (DOPG) and dioleoylphosphatidilcholine (DOPC) with a molar fraction of DOPG 0.3 (see details of the preparation and confirmation of BSMs, which is completely covered in the supplementary material Figure S1). The resulting mixture was vortexed for 30 min at the rate of 600 rpm; after that, the samples were rinsed three times with distilled water and five times with Tris buffer solution.

X-ray diffraction (XRD) measurements were carried out in a transmission mode using a URD-6 diffractometer (Carl Zeiss, Oberkochen, Germany). 

UV spectra were obtained by an Ultrospec 500/1100 pro spectrophotometer (Amersham, United Kingdom) in the range of 200–700 nm.

The spectrophotometric method was used to determine the capacitance of composites to doxorubicin. For this purpose, the solution of doxorubicin was added to the solution of the nanocomposites. The resulting mixture was dialyzed against water for 12 h. The external dialysis solution was analyzed by UV spectrophotometry. Solutions were placed in 1 cm glass cuvettes, and spectra were recorded in the wavelength ranges from 200–700 nm. The construction of the calibration plot for the determination of doxorubicin was realized using the peak at λ = 253 nm. The obtained value of the absorbance of DOX (at λ = 253 nm), ε = 1.42 × 10^5^ L×mole^−1^·cm^−1^, was used for calculating the concentration of doxorubicin.

Atomic-force microscopy imaging was performed using a scanning probe microscope Nanoscope IIIa (Veeco, Santa Barbara, CA, USA) operating in both contact and tapping modes in air. The cantilevers, made from silicon, were used (TipsNano, Zelenograd, Russia). The resonance frequencies were 140–150 KHz.

Sample preparation for AFM imaging. A droplet (20 µL) of micro gel suspension (0.1 wt. % concentration) was put onto a clean glass substrate modified with Ca^2+^, according to [23]. After 30 s of incubation, the unadsorbed particles were removed via spin-coating at 10,000 rpm. For AFM imaging, we used the so-called “soft tapping” regime, with delicate acting on the sample. The heights of the objects were calculated using Nanoscope software. The average sizes of 10–15 measurements were used. 

Hydrodynamic characteristics of the samples PNIPAM-PAA–β-FeO(OH) and PNIPAM-PAA–β-FeOOH–DOX were measured on a ZetaPlus (Brookhaven, Holtsville, NY, USA) analyzer in 0.15 M salt solutions at pH ~7 by the dynamic light scattering (DLS) method. Values of zeta-potential were obtained by laser microelectrophoresis on the ZetaPlus (Brookhaven, Holtsville, NY, USA) analyzer. For this purpose, aqueous solutions of the nanocomposite with a volume of 1.7 mL (C = 0.5 mg mL^−1^) in a Tris buffer solution (pH 7) were prepared and filtered, passing through a filter with a pore size of 0.5 µm.

IR spectra of the studied samples were recorded on a Specord M-80 IR (Carl Zeiss, Jena, Germany) spectrophotometer in the absorption measurement mode. The spectra were recorded in the wave number range of 400 to 2000 cm^−1^.

Cryo-TEM micrographs were taken on a Zeiss Libra 120 transmission electron microscope. The samples were vitrified in liquid ethane with the help of FEI Vitrobot Mark IV onto plasma-treated lacey carbon-coated grids. The vitrified specimens were transferred to a Gatan-910 cryo-holder. The images were recorded at a temperature of −170 °C, with an acceleration voltage of 120 kV.

The LUM-1 fluorescent microscope (Altami, Saint Petersburg, Russia) was used for the imaging of the interaction of model membranes with drug-loaded microgels.

A cytotoxicity MTT test was carried out on MCF7 cell culture. Cells were seeded in 96-well plates, 4000 cells per well, and were incubated with the substrate for 1 h in serum-free culture medium, each concentration in triplicate. Control wells contained no substrate. Then, the medium was replaced with a fresh portion supplemented with 10 wt. % = fetal bovine serum, and the cells were cultured for an additional 3 days. The relative amount of surviving cells was detected using the 3-(4,5-dimethylthiazol-2-yl)-2,5-diphenyltetrazolium bromide (MTT) assay [24].

**Figure 1:**
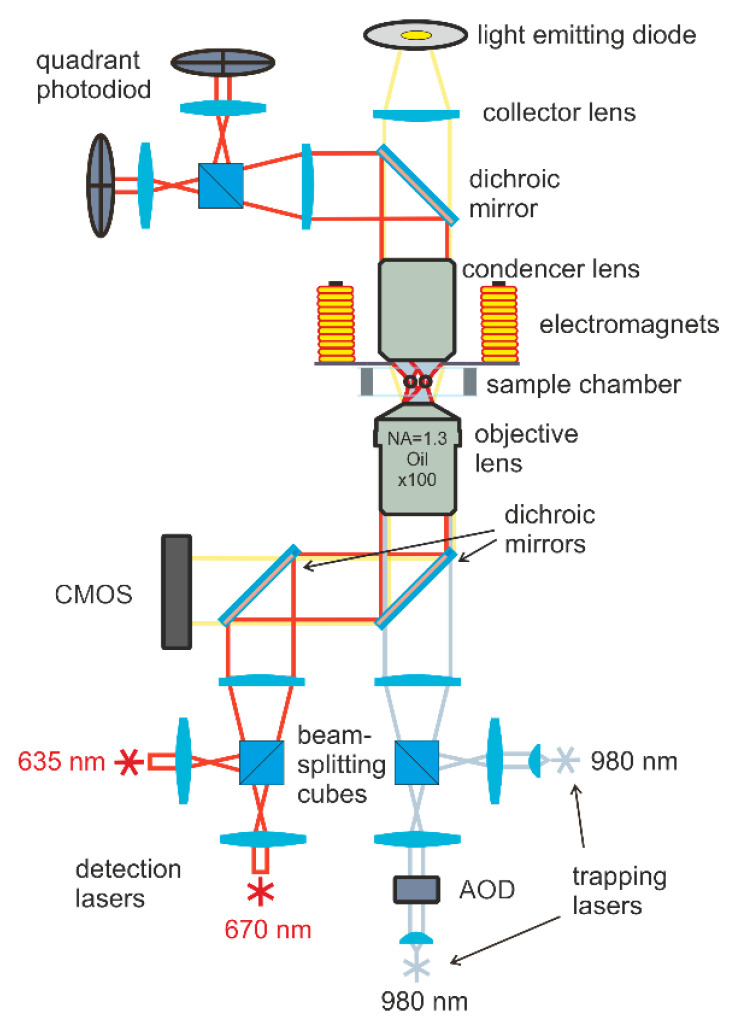
Double-trap optical tweezers scheme.

The magnetic moments of particles were measured using double-trap optical tweezers combined with electromagnets to apply a magnetic field to the trapped particles (see Figure 1). Two 980-nm diode lasers provide the trapping and manipulation of particles; then, the laser beams are tightly focused by an immersion objective with a numerical aperture (NA = 1.3). During the measurements, the laser power in the waist region was 5 mV. The positions of optical traps were controlled by the acousto-optical deflector (AOD) and steering lens system. Four electromagnets were located around the sample and made it possible to create a static or alternating magnetic field of 3 kA m^−1^ in the area of trapped particles.

The positions of the particles were determined using a detection system based on two additional lasers with wavelengths of 635 and 670 nm and a power of 0.1 mV. The radiation of these lasers passed through the particle and was collected on two quadrant photodiodes. The motion of the particles caused a change in the illumination of the diode and made it possible to detect the displacements of trapped particles with an accuracy of 10 nm. The sample was observed in transmitted light using an LED and CMOS camera. For more details of the double-trap optical tweezers setup and for the electromagnet arrangement scheme, see [25,26].

## 3. Results

**Figure 2:**
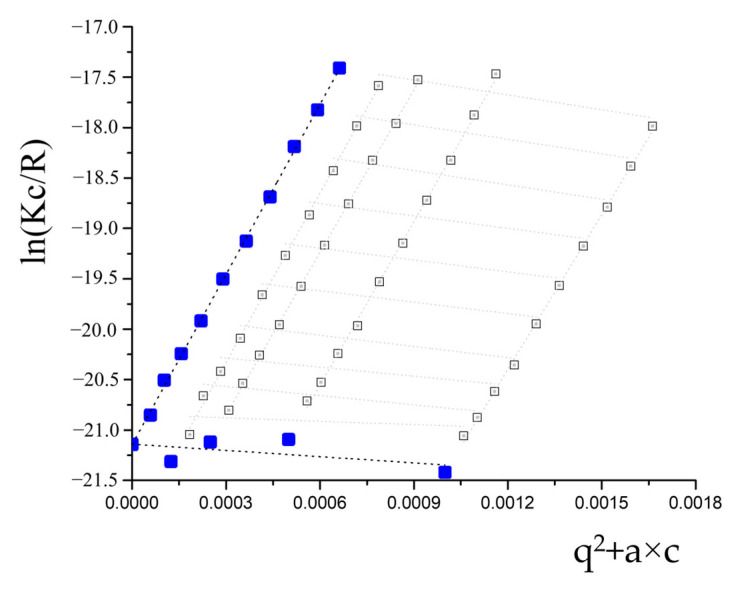
Guinier plot for the PNIPAM-PAA microgel. White squares correspond to experimental data, blue squares correspond to calculated approximations.

The analysis of the structure and morphology of the microgel was performed using a combination of static and dynamic light scattering. The results of static light scattering are presented in Figure 2 as a Guiner plot [27]. The molecular weight of the microgel was found to be Mw = 1.7 × 10^9^ Da, while the R_g_ was calculated to be 129 nm. The diffusion coefficient was estimated simultaneously, and the value of the hydrodynamic radius was calculated to be 180 nm. The R_g_/R_h_ ratio was found to be 0.72. For the hyperbranched macromolecules, it was demonstrated earlier by a combination of SAXS and light scattering that R_g_/R_h_ ratios below 0.778 correspond to the microgel structure [28]. Hence, the obtained R_g_/R_h_ ratio confirms the microgel structure of the synthesized PNIPAM-PAA [27,28,29].

**Figure 3:**
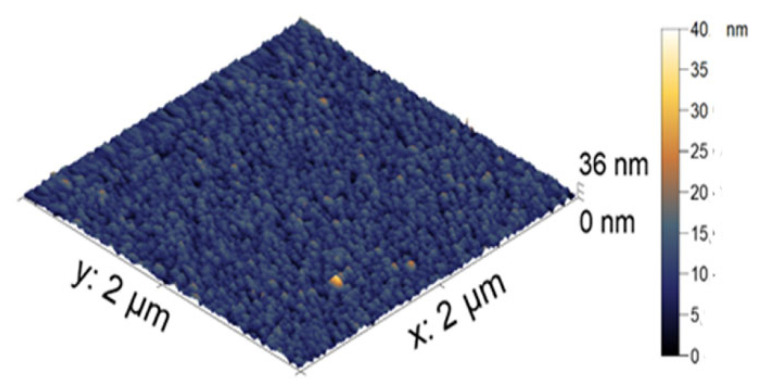
AFM images of the PNIPAM-PAA microgel.

The visualization of the microgel particles was made using AFM. In Figure 3, the AFM image of the microgel is presented as individual dried particles. The average height of the PNIPAM-PAA microgel was found to be 44 ± 2 nm. All microgel particles had a round shape. The difference in size in the hydrodynamic radius reflects two aspects. Dynamic light scattering gives enlarged sizes, while AFM produces understated values. The more important reason is that the hydrodynamic radius reflects the size of the microgel in a swollen state, and the AFM measurements in air give us the sizes of the contracted particles.

Nanocomposites were synthesized at room temperature via the treatment of a Mohr salt alkaline solution by sodium hypophosphite in the presence of the microgel PNIPAM-PAA. Earlier, it was found that the formation of nanocomposites takes place due to the synergetic actions of air, oxygen, and a strong reducing agent. It was shown that a reducing agent was necessary for the formation of metallic iron as an intermediate [30]. Without the reducing agent, a mixture of Fe^2+^/Fe^3+^ oxides and hydroxides were formed; however, it did not exhibit any pronounced magnetic properties.

To determine the concentration of Fe^3+^ in the obtained composite, it was completely deconstructed by concentrated sulfuric acid and then treated with sulfosalicylic acid. The resulting solution was analyzed spectrophotometrically according to a technique described elsewhere [30]. The content of Fe^3+^-ions was found to be 10.5 wt. %

The hydrodynamic radius of the nanocomposite was measured by dynamic light scattering and corresponded to 160 nm. 

**Figure 4:**
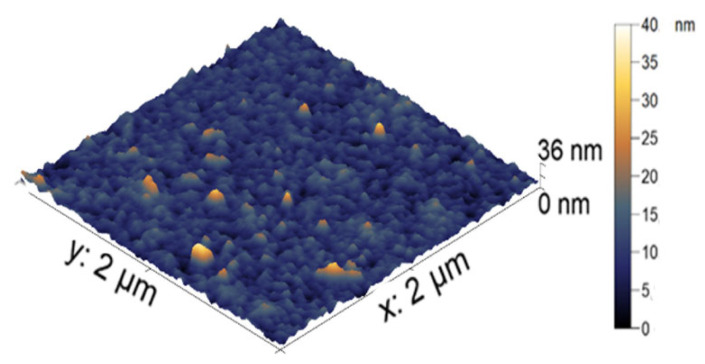
AFM images of the individual nanocomposite particles.

The visualization of the nanocomposite particles was made using AFM. In Figure 4, the AFM image of the nanoparticle-filled microgel is presented. The average height of the iron-modified PNIPAM-PAA microgel was found to be 19 ± 3 nm. All microgel particles had a round shape. In comparison with the initial PNIPAM-PAA microgel, a significant difference in size could be detected, reflecting the shrinking of the microgel particles due to additional crosslinking with nanoparticles. The difference in hydrodynamic radius reflects the difference between the swollen microgel and the contracted dried particles.

For information on the crystalline structure of the inorganic nanoparticles, the XRD technique was used. The XRD pattern of the investigated nanocomposite is shown in the supplementary materials (Figure S2). The XRD pattern consists of a set of reflections at 2θ = 30.85, 35.42, 42.84, and 57.53 degrees, similar to the data published in the literature [31]. The results obtained by the XRD method allow us to estimate that the structure of the nanoparticles corresponds to β-FeO(OH).

The chemical structure of the inorganic nanoparticles was identified using Mössbauer spectroscopy. ^57^Fe Mössbauer spectra of the modified microgel with η = 10.5 wt. % at 298 and 78 K (78 K) are shown in the supplementary materials (Figure S3). The spectrum at 298 K contains two doublets: internal (D1) and external (D2). The first, with a smaller line width W, corresponds to Fe^3+^ ions in an octahedral oxygen environment, e.g., in a [FeO_3_(OH)_3_] position. The environment of Fe^3+^-ions by H_2_O particles in the second position corresponds to the D2 doublet. The parameters of both doublets (Table 1) completely coincide with the literature data for synthetic bulky β-FeO(OH) [31,32].

The Mössbauer spectrum at 78 K is a superposition of several sextets at 474, 463, 459, and 436 kOe, with corresponding contributions of A of 20%, 20%, 11%, and 11% and the quadrupole doublet (A = 38%). The observed wide distribution of H values on ^57^Fe nuclei is determined by the significant heterogeneity of the particle size distribution of the sample. The 5–20 nm β-FeO(OH) particles are a reason for the unresolved magnetic hyperfine structure. The appearance of a quadrupole doublet means that the sample contains a fraction of small (presumably 1–3 nm) β-FeO(OH) particles [33].

The PNIPAM-PAA microgels were reported to demonstrate pH and temperature sensitivity [29]. Hence, the behavior of the PNIPAM-PAA-β-FeO(OH) at different temperatures and pH of the surrounding media was investigated. No significant change in the size of the composite microgel was detected in the temperature range from 20 to 45 °C. It seems that nanoparticles form rigid frameworks inside the microgel [34] that prevent shrinking. The results of measurements of the zeta-potential of the composite microgel at the pH range from 9 to 5 demonstrated no change in the surface charge of the microgel in basic, neutral, and slightly acidic media (see Table 2). At pH 5, the loss of solubility of the microgel and phase separation was registered. 

**Figure 5:**
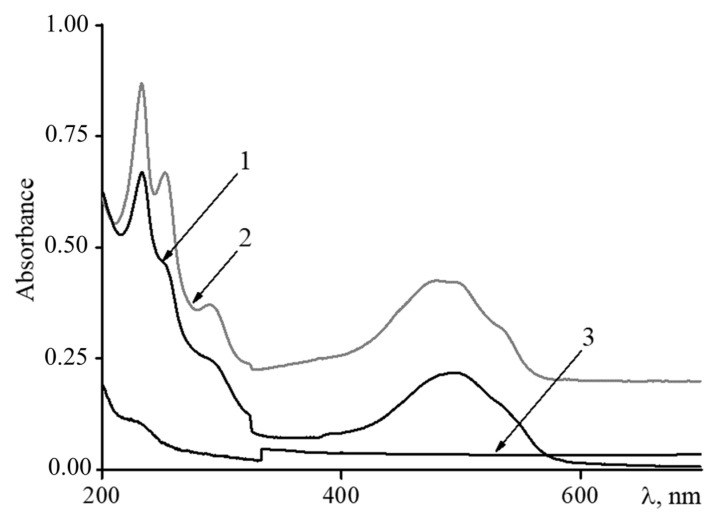
UV spectra of (1) triple nanocomposite NIPAM-PAA-β-FeO(OH)-DOX, [DOX]/[AC] = 1/1; (2) pure doxorubicin; in all cases CDOX = 3 × 10^−5^ M; (3) external dialysis solution after interaction of DOX and NIPAM-PAA-β-FeO(OH).

The loading of DOX into the composite microgels was carried out by varying the ratio of DOX to acrylic carboxyl (AC) groups in the nanocomposite ([DOX]/[AC]). The purification of drug-loaded composites from unbound DOX was carried out by dialysis. Binding control was performed by analyzing both the external dialysis solution and the internal solution by UV spectroscopy. Typical spectra are presented in Figure 5. 

The UV spectrum of DOX displays bands at 288 and 480–500 nm, corresponding to the two allowed transitions, polarized along the short and long axes of the anthracycline fragment, respectively [35]. A shoulder around 320–380 nm is associated with n→π transitions of the C=O groups in the molecule, partially forbidden by electric dipole [36]. At the described conditions (pH 7.4), the aglycone part of DOX is neutral, whereas the daunosamine part is in the protonated form [37]. Hence, bands at 252 and 233 nm have been assigned to the aglycone moiety, with the contribution of the protonated daunosamine moiety [38,39].

After interaction with the magnetic microgels in the DOX spectrum, a significant drop in the absorption intensity at 232 and 255 nm takes place.

This result evidently shows, first, the electrostatic interaction of DOX (through protonated amino groups) and the magnetic nanocomposite (through charged carboxylate groups), and second, the formation of a ternary complex, PNIPAM-PAA-β-FeO(OH)-DOX, in which DOX molecules are incorporated in the magnetic microgels. It was found that the maximum DOX loading corresponds to a [DOX]/[AC] ratio of 3/1. The incorporation of DOX in the nanocomposite was found to be quantitative—no DOX was found in the external dialysis solution Figure 5 curve 3.

**Figure 6:**
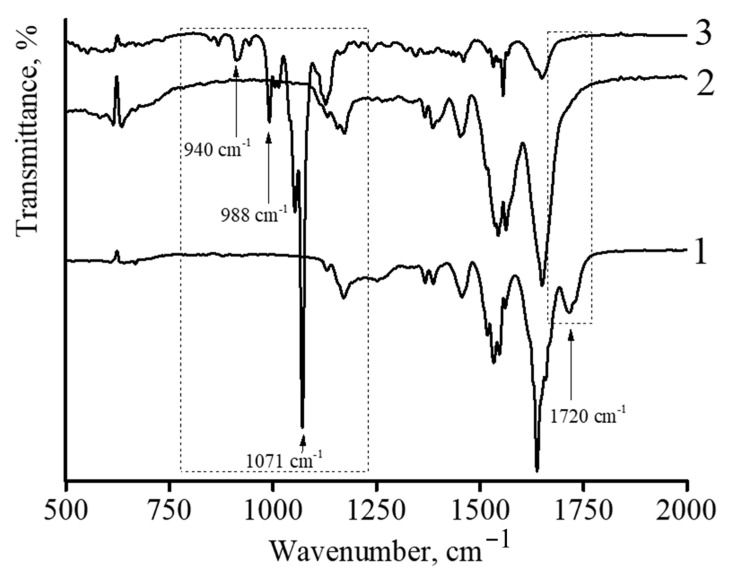
IR spectra of polymer microgel NIPAM-PAA (1); nanocomposite NIPAM-PAA-β-FeO(OH) (2); triple nanocomposite NIPAM-PAA-β-FeO(OH)–DOX, [AC]/[DOX] = 1/1 (3).

IR spectroscopy was also used to characterize DOX-loaded nanocomposites. Figure 6 shows the infrared spectra of described microgels. In the case of the initial microgel based on NIPAM-PAA, the following characteristic peaks were observed (Figure 6 curve 1). The amide characteristic peaks at the wavenumbers of 1650 and 1540 cm^−1^ can be assigned to C=O (amide I) and N-H bonding (amide II), respectively [40,41]. The strong absorption band at 1720 cm^−1^ belongs to the carbonyl group of PAA. The spectra of the microgel showed the characteristic amide groups at 1650 and 1540 cm^−1^ of PNIPAM [42,43]. Furthermore, the peaks observed at 1455 and 1380 cm^−1^ suggested the presence of -CH_2_ and -CH_3_ groups in the microgels, respectively. No peaks corresponding to non-saturated C=C bonds were found in the spectrum, reflecting the complete conversion of monomers in microgel synthesis.

In the case of the NIPAM-PAA-β-FeO(OH) nanocomposite (Figure 6 curve 2), a sharp decrease in the peak at 1720 cm^−1^, which belongs to the carbonyl group of PAA, is observed. In addition, the spectrum of the microgel NIPAM-PAA-β-FeO(OH) contains a peak at 848 cm^−1^, which is characteristic of the β-FeO(OH) phase [44,45].

In the case of the NIPAM-PAA-β-FeO(OH)–DOX microgel (Figure 6 curve 3), besides all peaks being characteristic for the NIPAM-PAA-β-FeO(OH) microgel, peaks at 988 and 1010 cm^−1^ (C–O stretch, primary alcohol), 1071 cm^−1^ (C–O stretch, secondary alcohol), 938 cm^−1^ (C–O–C stretch), and 916 cm^−1^ (bending vibration of C–OH), characteristic of doxorubicin [27,29], are presented in the IR spectrum. It is important to stress that microgels without DOX have no such peaks at these wavelengths.

The obtained results indicate that the stabilization of β-FeO(OH) nanoparticles occurs due to complexation with the carboxyl groups of PAA in the composition of the NIPAM-PAA microgel. Furthermore, the microgel based on NIPAM-PAA-β-FeO(OH) is able to bind DOX, as confirmed by UV spectroscopy data.

The effect of the loading of DOX on the colloidal stability of the microgel was investigated by dynamic light scattering and laser-microelectrophoresis methods. The hydrodynamic radius of the PNIPAAM-PAA-β-FeO(OH)-DOX microgel was found to be 200 nm. Zeta-potentials of the obtained microgels were measured by laser microelectrophoresis. The results are presented in Table 3. All microgels, including the initial NIPAM-PAA, had zeta-potential values in a region from −(28 ± 2) mV. This result indicates the colloidal stability of the prepared nanocomposites.

**Figure 7:**
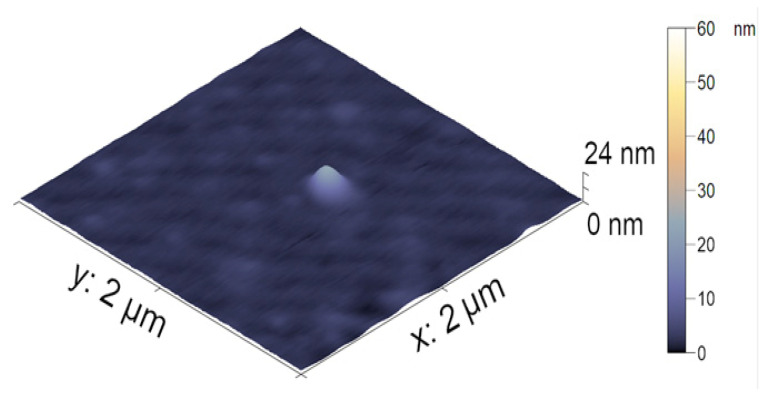
AFM images of the NIPAM-PAA-β-FeO(OH)–DOX microgel.

In order to demonstrate the influence of DOX incorporation on the morphology of the composite microgel, AFM imaging was performed. The particle of the DOX-loaded magnetic microgel is presented in Figure 7. The incorporation of DOX in the microgel did not result in a change in the spherical shape of the particle. The average height of the DOX-loaded NIPAM-PAA-FeO(OH)-DOX was measured to be 12 ± 1 nm. Additional contractions in comparison with the size of the NIPAM-PAA-FeO(OH) microgel should be mentioned. Thus, the absorbance of DOX molecules into the microgel results in additional contractions of the particles.

**Figure 8:**
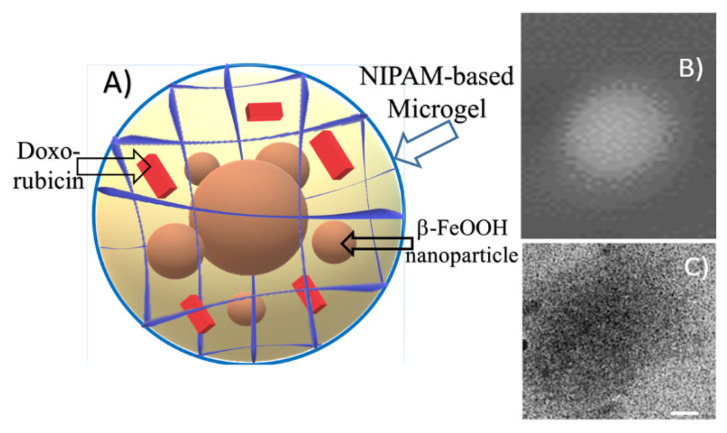
NIPAM-PAA-β-FeO(OH)-DOX structure—schematical representation (**A**), AFM (**B**), and Cryo-TEM images (**C**). White bar on the left upper image corresponds to 40 nm.

Additional information about DOX-loaded microgel morphology was obtained via Cryo-TEM imaging. In Figure 8, the scheme of the composite particle is presented. A typical microphotograph of the drug-containing microgel is shown in Figure 8C. Contrast quasi-spherical particles with sizes within the 150-250 nm range could be observed in the image. These data are in good agreement with hydrodynamic size measurements. According to the similar diameter of particles obtained by DLS, AFM, and Cryo-TEM, we can refer to these particles as dense spherical particles.

The measurements with optical tweezers gave the cumulative magnetic moment of the NIPAM-PAA-β-FeO(OH) microgel of 1.3 ± 0.6 fA × m^2^ in an external magnetic field of 62 Oe. To obtain the magnetic moment, 45 μL of an aqueous microgel suspension was placed in a hermetic chamber, which consisted of two cover glasses and a 0.15 mm thick sealing gasket between them. The chamber was placed on a mechanical stage of an optical tweezers setup, above the objective lens that creates optical traps. Two particles were captured in two independent optical traps separated by distance R. Both traps were located at 15 μm above the bottom cover glass to minimize interactions with the medium boundary. The measurements were carried out at a temperature of 20 °C, so the particles were displaced from the centers of the traps under the action of thermal fluctuations. Analysis of the Brownian motion of trapped particles allows the determining of the characteristics of an optical trap, namely, the stiffness of the optical trap *k*, which is the proportion coefficient between an optical trap restoring force and a particle’s displacement from the center of the trap [26]. Periodic displacement of the position of the traps with an amplitude of *A_trap_* causes the trapped particles to oscillate with an amplitude *A_osc_*, which depends on the stiffness of the optical trap, viscous friction, and oscillation frequency. Periodic displacements of particles can be obtained in another way based on their magnetic interaction. Then, an alternating magnetic field was directed along the axis connecting the centers of the particles; a magnetic moment was periodically induced in the particles. The interaction of the magnetic moments of particles led to an attraction between particles and to their displacement from the centers of optical traps, with a frequency coinciding with the frequency of the absolute value of the external alternating magnetic field. A comparison of amplitudes of displacements due to magnetic interaction *A_magn_* and due to trap position oscillation *A_osc_* made it possible to determine the magnitude of the magnetic moments of particles in a specific external magnetic field, such as the following equation:

(2) $$m=\sqrt{\frac{4\pi R^{4}}{3\mu_{0}}\frac{A_{magn}}{A_{osc}}kA_{trap}}.$$

**Figure 9:**
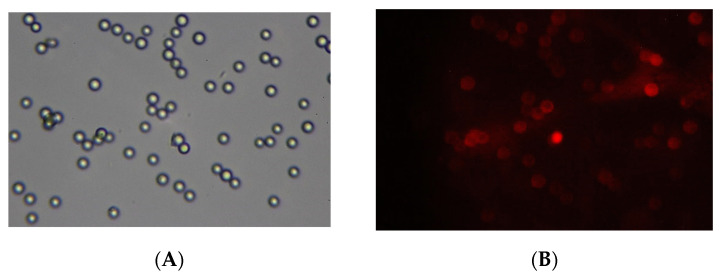
Images of lipid-bilayer-covered BSLMs incubated with NIPAM-PAA-FeO(OH)–DOX in the presence of a magnet. Optical channel (**A**) and fluorescent channel (**B**).

A demonstration of the magneto-controlled delivery of microgels to biological membranes was made as follows. The NIPAM-PAA-FeO(OH)–DOX solution was added to a suspension of sedimented 2 mg of BSM covered with a lipid bilayer (BSLM) in a test tube. The neodim magnet was placed under the resulting composition. After 15 min of incubation, the solution with unbound NIPAM-PAA-FeO(OH)–DOX was disposed and the sediment was rinsed with Tris buffer solution. Finally, the BSLMs were resuspended in Tris buffer and transferred to a glass slide in order to take pictures using a fluorescent microscope. The images are presented in Figure 9. In the optical channel, one can see the number of BSLM particles. In the fluorescent channel, the color red, corresponding to the fluorescence of DOX, is observed on these BSLM particles reflecting the adsorption of NIPAM-PAA-FeO(OH)–DOX particles on the surface of the biomimetic membranes. Thus, the magnetic field could be an effective stimulus to govern the adsorption of anionic magnetic microgels on the desired biomembranes.

Finally, the cytotoxicity of the NIPAM-PAA-FeO(OH)–DOX was studied by an MTT test. Recently, PNIPAM-PAA microgels have been reported to be nontoxic [14]. It was found that the concentration of DOX in the NIPAM-PAA-FeO(OH)–DOX microgel that results in the death of 50% of cells (IC50) was equal to 1 μM. In comparison, the IC50 of DOX in a microgel-free system was found to be 0.8 μM. Thus, the immobilization of DOX molecules in magnetic microgel does not result in the loss of its anticancer activity. It is important to stress that in the control experiment, the DOX-free NIPAM-PAA-FeO(OH) microgel was found to have no cytotoxic effect in a wide range of concentrations. Hence, the NIPAM-PAA-FeO(OH) microgel could serve as nontoxic vehicle for the magnetic-driven controlled delivery of bioactive substances. 

## 4. Discussion

A one-pot synthesis of magnetically controlled nanocontainers based on NIPAM-PAA polymer could be carried out at room temperature by the direct synthesis of nanoparticles in solution with microgels. Nanoparticles’ growth in the microgel matrix restricts their size, preventing precipitation. Among the possible crystalline structures of iron oxide, obtaining in the presence of stabilizing polyacrylic acid groups results in the formation of the magnetic phase of β-FeO(OH) nanoparticles which was confirmed by XRD and Mössbauer spectroscopy. One of the key properties of these magnetic nanoparticles is their low reactivity and low toxicity. The size of the nanocomposites was determined both by visualization methods (Cryo-TEM, AFM) and using the light scattering method. It was established that the size of magnetically controlled nanocontainers does not exceed 250 nm in diameter, and the electrokinetic characteristics indicate a high negative value of the zeta-potential of nanocontainers, reflecting high colloid stability. At the same time, the incorporation of 10 wt.% of magnetic nanoparticles into PNIPAM-PAA microgel resulted in the loss of the thermosensitivity of the microgel in a wide range of temperatures. Due to the electrostatic interaction of iron-oxide nanoparticles and carboxylate groups, the additional crosslinking of the anionic microgel can be observed [34]. As a result, a rigid framework is formed. The surprising result of the incorporation of magnetic nanoparticles on the pH sensitivity of the microgel could be explained as follows. Magnetic nanoparticles induce the additional deprotonation of the carboxyl groups. The shift of the protonation-deprotonation equilibrium for the weak polyelectrolytes was reported earlier for the polyacrylic acid interacting with opposite-charged polyelectrolytes and colloids [46,47]. Recently, we have demonstrated that at neutral pH for the composite of a weak polyanion and the magnetic nanoparticles, an increase in the ratio of nanoparticles in the microgel results in a significant increase in the surface charge of the microgel [48]. 

The results, obtained by means of laser optical tweezers, deserve special attention. These results allowed us to determine the value of the magnetic field, sufficient for the possibility of manipulation by individual particles of a polymeric magnetic carrier. The obtained results indicate, first, the high colloidal stability of nanocontainers, and second, the possibility of its wide biomedical application and the implementation of targeted drug delivery.

High response to the magnetic manipulations demonstrates the possibility of using magnetic nanocontainers for the controlled delivery of the drug doxorubicin. While anionic microgel is responsible for the incorporation of the drug, the magnetic particles ensure the external magnetic field-driven targeted delivery. The size of nanocomposites filled with doxorubicin was determined by both the visualization method (Cryo-TEM, AFM) and using the light scattering method. It has been established that loading with doxorubicin does not significantly affect either the hydrodynamic or electrokinetic characteristics of the resulting ternary systems. Hence, we may suggest that the accumulation of the doxorubicin takes place in the internal volume of the microgel but not on the surface. As biomembranes carry a net negative surface charge, it is important to overcome the electrostatic repulsion between the membrane and the negatively charged drug-loaded microgel. The application of the magnetic field is capable of overcoming this repulsion, and the anionic microgel can easily reach the surface of the biomimetic membrane.

## 5. Conclusions

Anionic magnetic microgels loaded with the antitumor drug doxorubicin, NIPAM-PAA-FeO(OH)–DOX, were obtained. The morphology of the β-FeO(OH) magnetic phase was confirmed by the data from X-ray analysis, IR spectroscopy, and transmission electron microscopy. Using the technique of dynamic light scattering, it was found that in isotonic solutions, the particle size of DOX-free microgels NIPAM-PAA-FeO(OH) is 200 nm. Magnetic microgels loaded with doxorubicin are characterized by similar sizes. In addition, by means of laser microelectrophoresis, both NIPAM-PAA-FeO(OH) microgels and magnetic doxorubicin-loaded magnetic microgels are characterized by high colloidal stability. It was shown that NIPAM-PAA-FeO(OH) microgels quantitively bind the DOX-drug. The optical tweezers study of the microgels has demonstrated the pronounced magnetic properties of individual microgel particles and, therefore, the possibility of manipulation with NIPAM-PAA-β-FeO(OH)–DOX particle traffic using altering magnetic fields. These results demonstrate the advantages of the suggested system for targeted drug delivery.

## Figures and Tables

**Table 1:** Parameters of Mössbauer spectrum for iron-modified microgel with *η* = 10.5 wt. % at 298 K.

Component	δ, mm/s ±0.01	ΔE, mm/s ±0.01	W, mm/s ±0.01	A, % ±0.03
D1	0.36	0.55	0.35	56
D2	0.35	0.90	0.42	44

δ—isomer shift; ΔE—quadrupole splitting; W—full width at half height; A—relative contribution (relative subspectra area).

**Table 2:** The values of zeta-potential of the PNIPAM-PAA-β-FeO(OH) microgel at different pH values.

pH	9	7	6	5
Zeta-potential	−38.2 ± 2.92	−36.92 ± 3.73	−36.36 ± 4.10	Not applicable *

* Phase separation of the system.

**Table 3:** Electrokinetic characteristics of magnetic microgels based on NIPAM-PAA-FeO(OH) and doxorubicin-loaded magnetic microgels.

(Fe^3+^), wt. %	Z-Potential of Composites NIPAM-PAA-β-FeOOH, mV	Z-Potential of Composites NIPAM-PAA-β-FeOOH-Doxorubicin, mV
10.5	−(27.4 ± 1.5)	−(28.7 ± 1.3)

## Data Availability

The data presented in this study are available on request from the corresponding author.

## Supplementary Materials

The following supporting information can be downloaded at: https://www.mdpi.com/article/10.3390/polym14245440/s1, Figure S1: Images of the BSM and BSM covered with DOPC/DOPG/Rh-DOPE bilayer in optical and fluorescent microscope; Figure S2: The XRD-pattern of the iron containing nanocarrier based on PNIPAM-PAA; Figure S3: Mössbauer spectrum of iron-containing nanocarrier based on NIPAM-PAA.
