# Supplementary material for: Synthesis of Magneto-Controllable Polymer Nanocarrier Based on Poly(N-isopropylacrylamide-co-acrylic Acid) for Doxorubicin Immobilization
Source: Polymers (Basel). 2022 Dec 12;14(24):5440. doi: 10.3390/polym14245440 (PMC9784437; doi:10.3390/polym14245440)
Supplement: Supplementary file 1 [file polymers-14-05440-s001.zip › polymers-2068399-supplementary.pdf]

polymers
MDPI

Article

Synthesis of Magneto-Controllable Polymer Nanocarrier Based on Poly(N-isopropylacrylamide-co-acrylic Acid) for Doxorubicin Immobilization

Viktoria S. Kusaia [1], Elena Yu. Kozhunova [1,2,*], Darya A. Stepanova [1], Vladislava A. Pigareva [1], Andrey V. Sybachin [1], Sergey B. Zezin [1], Anastasiya V. Bolshakova [1], Nikita M. Shchelkunov [2], Evgeny S. Vavaev [2], Evgeny V. Lyubin [2], Andrey A. Fedyanin [2] and Vasiliy V. Spiridonov [1]

Small unilamellar liposomes were prepared by sonication technique. The weighted amounts of electroneutral dioleoylphosphatidylcholine (DOPC) and anionic dioleoylphosphatidylglycerol (DOPG) were dissolved in methanol/chlorophorm mixture so that the molar fraction of the anionic lipid [DOPG]/ ([DOPC]+[DOPG]) was 0.3. Then the organic solvent was evaporated on Laborota vacuum rotor evaporator. The resulted thin lipid film was dispersed in 0.01 M Tris buffer with a pH of 7.0. The freshly formed suspension was subjected to sonication of Cole-Parmer CP-750 tip ultrasonicator for the two 5-min cycles. The titanium tip dust was separated from the sample of the prepared liposomes by centrifugation on Eppendorf Mini-spin centrifuge with 10 000 rpm for the 5 min. The average size of the liposomes was 50 nm. The average electrophoretical mobility of the liposomes was -3 (μm/s)/(V/cm). The samples were used within one day after preparation.

Biomimetic lipid membranes (model cell membranes) were prepared by the following technique. The 2 mg of 5 microns borosilicate beads from Duke Scientific (USA) were dispersed on 1 ml of ethanol and then separated from ethanol by centrifugation. Washed with ethanol beads were dispersed in 1 ml of 1 M KOH solution and then separated by centrifugation. Washed beads were rinsed with DI water and Tris buffer to clean the surface. Then 2 mg of beads in 1 ml Tris buffer were mixed with suspension of freshly prepared by sonication technique small unilamellar anionic DOPG/DOPC liposomes with molar fraction of DOPG 0.3 (concentration of liposomes was 0.4 mg/ml) on Biosan vortex with of 600 rpm during 30 min. Then non-reacted liposomes were separated by centrifugation and the beads with supported lipid membrane (BSLM) were obtained.

The 100% coverage of the beads surface was controlled with the use liposomes labeled with rhodamine modified lipid dioleoylphosphoethanolamine (Rh-DOPE). The images of BSLMs in optical microscope and fluorescent microscope (using the filter with λ_{ex} = 460-550 nm) are presented on Figure S1. No fluorescent signal was detected for uncovered microspheres. For the BSLMs the red fluorescence of the whole surface of the beads was observed.

Materials

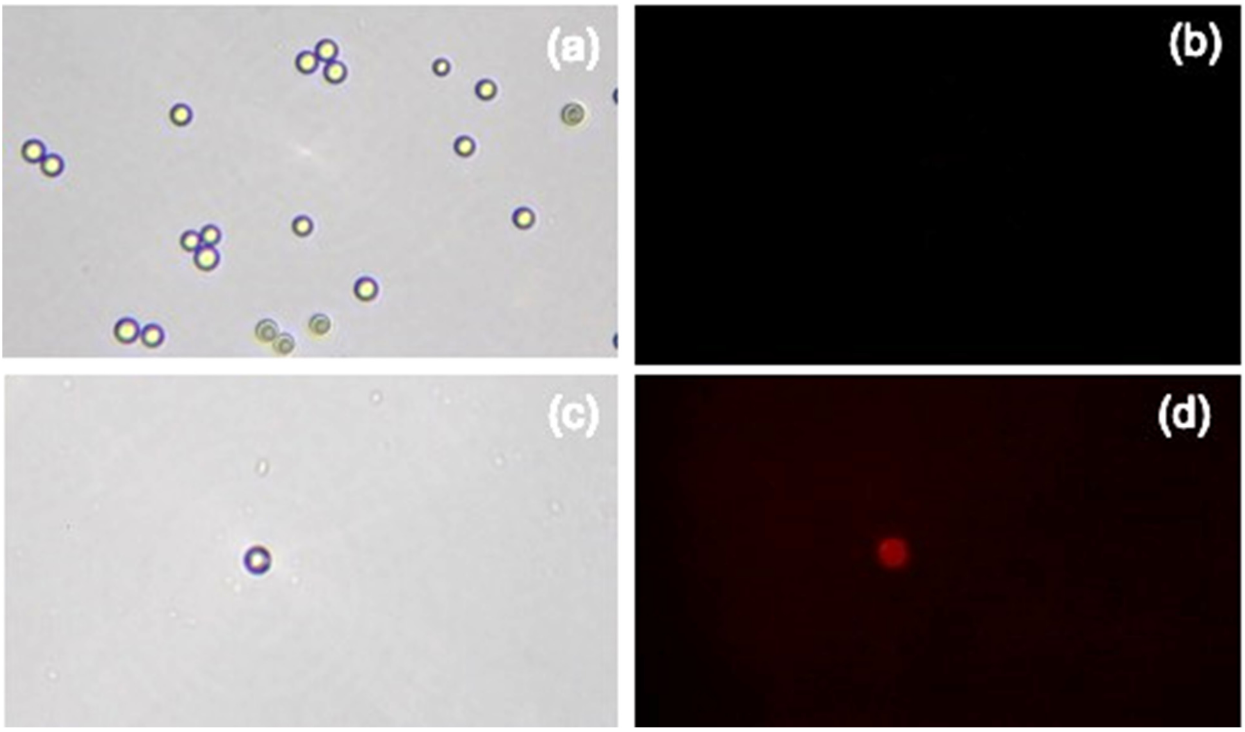

Figure S1. Images of the initial borosilicate beads (a,b) and covered with DOPC/DOPG/Rh-DOPE bilayer (c,d) in optical (a,c) and fluorescent microscope (b,d). Fluorescence excitation filter with λex = 460-550 nm (b,d).

Results

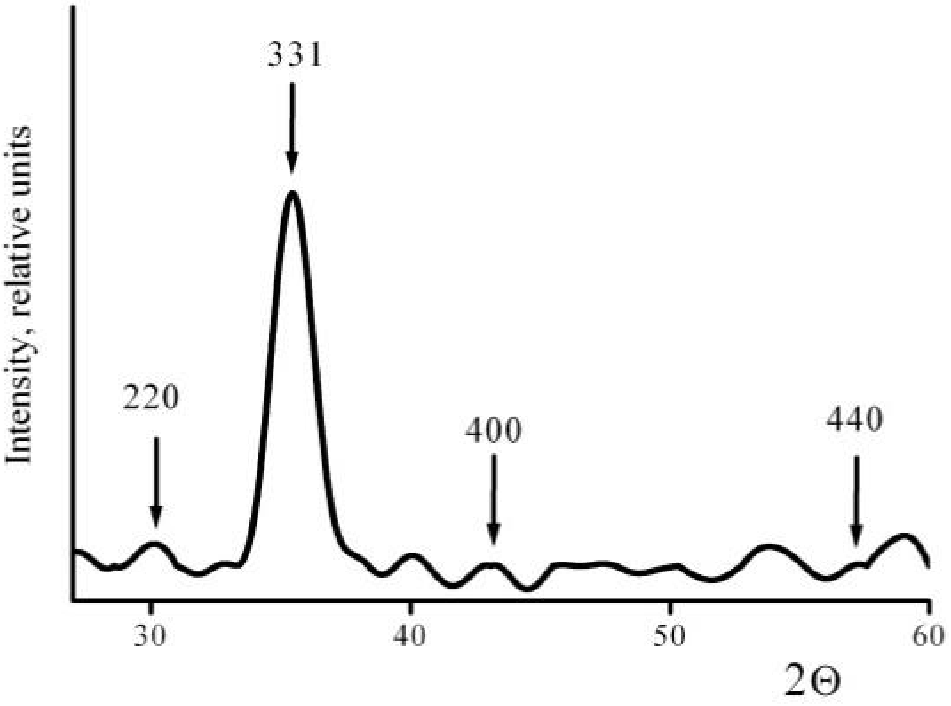

Figure S2. The XRD-pattern of the iron containing nanocarrier based on PNIPAM-PAA.

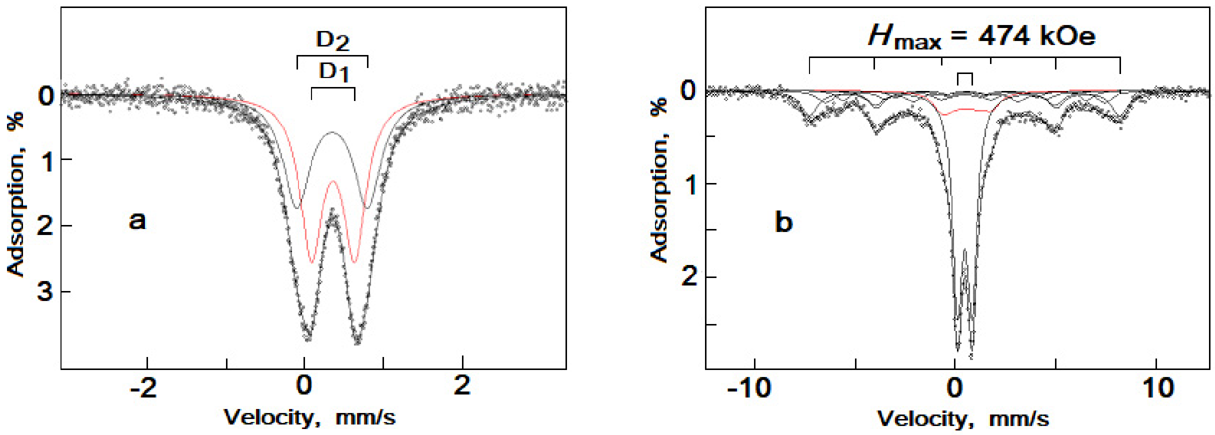

Figure S3. Mössbauer spectrum of iron-containing nanocarrier based on NIPAM–PAA.
